# Analytical Comparison of Risk Prediction Models for the Onset of Macrosomia Based on Three Statistical Methods

**DOI:** 10.1155/2022/9073043

**Published:** 2022-09-10

**Authors:** Jinbo Zhang, Xiaozhi Wu, Qingqing Song

**Affiliations:** ^1^Department of Obstetrics and Gynecology, The Third Affiliated Hospital of Zunyi Medical University (The First People's Hospital of Zunyi), Zunyi, 563000 Guizhou, China; ^2^Department of Obstetrics, The First Affiliated Hospital of Zunyi Medical University, Zunyi, 563000 Guizhou, China

## Abstract

**Method:**

A retrospective selection of 93 women who were hospitalized in our hospital from March 2019 to May 2022 with a singleton pregnancy and delivered at term with macrosomia were the study group. And 356 women who delivered a normal size baby during the same period were the control group. The variables that were associated with the onset of macrosomia were screened from maternal medical records. Logistic regression models, random forest, and CART decision tree models were developed using the screened variables as input variables and whether they were macrosomia as outcome variables, respectively. The performance of the three models was evaluated by accuracy, precision, recall, F1 score, and receiver operating characteristic curve (ROC).

**Result:**

The risk prediction models for the onset of macrosomia, logistic regression model, random forest model, and decision tree, were successfully developed, with accuracies of 0.904, 1.000, and 0.901 in the training set and 0.926, 0.582, and 0.852 in the validation set, respectively. The AUC in the training set were 0.898, 1.000, and 0.789, and in the validation set were 0.906, 0.913, and 0.731, respectively. In general, the logistic regression model has the highest diagnostic efficiency, followed by the random forest model.

**Conclusion:**

Logistic regression models have high application value in the assessment of predicting the risk of macrosomia, and it is suggested that the advantages of logistic regression models and random forest models should be combined in future studies and applications to make them work better in the prediction of the risk of macrosomia.

## 1. Introduction

Macrosomia (weight ≥ 4000 g) is a common obstetric complication. Data show that the incidence of macrosomia abroad in the past decade is about 15.1% [1]. However, the incidence of macrosomia varies greatly among different regions in China, averaging about 7%-9% [2, 3]. Neonatal weight affects the delivery mode of pregnant women and can increase the risk of assisted delivery, dystocia, neonatal birth injury, birth canal injury, and other risks for vaginal delivery, as well as the incidence of neonatal asphyxia [4, 5]. At present, the mechanism of macrosomia is not clear, and the occurrence of macrosomia is usually the result of multiple factors [6].

With the improvement of health care, although the incidence of macrosomia has started to show a decreasing trend globally, it is still a public health issue of wide concern because of its more serious impact on maternal and infant safety. At present, clinical prediction mainly relies on obstetric investigations and imaging [7–9], but their accuracy is still controversial. There is no accepted method to accurately predict macrosomia. Although most of the current studies on risk factors for macrosomia are given risk factors and single confidence intervals, there are not many reports on methods to establish risk prediction. In recent years, machine learning has been increasingly used in healthcare, with decision tree models that can handle predictive models with nonlinear relationships and random forest (RF) models that are more robust and can efficiently handle large data sets [10, 11]. Therefore, in this study, we retrospectively analyzed maternal related data from the maternal health database in the Department of Obstetrics and Gynecology of our hospital, and proposed to construct a prediction model for the risk of occurrence of macrosomia by retrospectively using the multifactor logistic regression (LR), RF, and CART algorithm decision tree (DT). Then, we compared the prediction performance of the three models to provide technical support and theoretical basis for prediction and prevention of huge children.

## 2. Data and Methods

### 2.1. Research Data

The puerperas who were hospitalized in our hospital from March 2019 to July 2022 with a singleton pregnancy and delivered macrosomia at term were retrospectively collected as the study group. The puerperas who were hospitalized during the same period and delivered a normal size baby (weight ≥ 2 500 g and<4 000 g) were randomly selected as the control group. Both groups were excluded according to the following criteria: (1) puerperas have history of diseases such as polycystic ovary syndrome, diabetes, and hypertension before pregnancy; (2) puerperas have heart, liver, lung, kidney, and other important organ diseases; (3) puerpera is through in vitro fertilization, assisted reproduction; (4) puerperas have benign or malignant tumors (including uterine fibroids and ovarian cysts); (5) puerperas taking drugs affecting glucose and lipid metabolism before pregnancy; and (6) puerperas who have incomplete data or do not agree to use the data for the researcher. Finally, 93 women with macrosomia were included in the study group and 356 women in the control group. This study was approved by the Ethics Committee of The Third Affiliated Hospital of Zunyi Medical University (The First People's Hospital of Zunyi).

### 2.2. Methods

The following information was collected by trained subject members by reviewing electronic and paper medical records in the hospital case room: (1) basic information: age, height, prepregnancy body mass, prepregnancy BMI, total weight gain during pregnancy, number of pregnancies and deliveries, etc. (2) Laboratory test results (blood count, blood glucose level, lipid level, thyroid function, etc.). (3) Pregnancy status: whether the pregnancy is combined with gestational diabetes or gestational hypertensive disease. (4) Delivery outcome: information on the birth quality and sex of the newborn.

### 2.3. Variable Definition

Pregnancy BMI = pregnancy weight (kg)/height (m)^2^. According to different prepregnancy BMI strata, the BMI intervals were based on the Chinese adult BMI classification, i.e., normal (18.5 kg/m^2^ ≤ BMI < 24.0 kg/m^2^), underweight (<18.5 kg/m^2^), overweight (24.0 kg/m^2^ ≤ BMI < 28.0 kg/m^2^), and obese (≥28.0 kg/m^2^). Pregestation weight was defined as the last measured body mass after admission. Gestational weight gain = Pregestation weight-prepregnancy weight. Gestational diabetes is diagnosed according to the following criteria: a 75 g oral glucose tolerance test (OGTT) is performed at 24 to 28 weeks of gestation, and blood glucose values are measured on fasting and 1 and 2 h after taking glucose, respectively. Fasting blood glucose values ≥ 5.1 mmol/L, or 1 h ≥ 10.0 mmol/L, or 2 h ≥ 8.5 mmol/L. Gestational diabetes is diagnosed if any of the above three indicators are met. Hypertensive disease of pregnancy (HDP) includes gestational hypertension, preeclampsia-eclampsia, chronic hypertension (caused by any causes diagnosed before 20 weeks of pregnancy), and chronic hypertension with preeclampsia [12].

### 2.4. Model Building

#### 2.4.1. Establishment of the LR Model

First load the library (rms) and import the data in txt format. Then, 70% of the data are randomly selected as the training set and 30% as the validation set. The LR model is constructed under the glm function using the training set. Finally, the LR model is filtered by stepwise regression of AIC criterion using the step function.

#### 2.4.2. Establishment of the Decision Tree Model

The DT uses the CART algorithm, which consists of feature selection, tree generation, and pruning, to solve binary classification problems. DT generation is the process of recursively constructing a binary decision tree. The Rpart software package is used for automatic computation, and the Gini coefficient minimization criterion is used to select features, generate a binary tree containing root, internal and leaf nodes, and automatically compute the estimation error identification complexity parameter for cross-validation.

Model construction process: after importing data, install and load the library (rpart) package for subsequent use. Random sampling is done to set the seeds, then 70% of the data are randomly selected for the training set and 30% for the validation set. Then, we build the decision tree and check the classification tree information, and finally output the results.

#### 2.4.3. Establishment of the Random Forest Model

The construction of the RF classification and recognition model is performed by calling the random forest package in the R Studio environment. The modeling process of RF contains two important parameters: ntree (the number of decision trees in the RF algorithm) and mtry (the number of variables used to set the branches of the decision tree in the RF algorithm). The square root of the number of variables in the data set is usually used as the value of the mtry parameter. Ntree is usually set to 500, i.e., there are 500 trees in the RF algorithm by default. According to the above default parameters, the initial RF algorithm model is built, the classification model effect is evaluated in the test set, and the optimal parameters are selected to build the final model.

Model construction process: after importing data, library (randomForest) is loaded with the random forest package. Random sampling is used to set the seeds, and then 70% of the data are randomly selected for the training set and 30% for the validation set. Then, use the training set data to build the RF model.

### 2.5. Statistical Processing

Epidata 3.0 software was used to establish the database. Statistical analysis was performed using the R Foundation for Statistical Computing, Vienna, Austria version 4.0.3. Variables with statistically significant differences were screened out by single factor analysis to establish the LR model, RF algorithm model, and DT algorithm model, respectively. 70% samples were selected from the study group and the control group, respectively, to form a training set for building the model, and the remaining 30% samples were used as test sets to evaluate the model performance. The models were compared by calculating the accuracy, precision, sensitivity, specificity, recall, F1 score, and area under the receiver operating characteristic curve (AUC) values of the three models. The *P* value less than 0.05 was considered as a statistically significant difference.

The calculation formula of accuracy rate, precision rate, recall rate, and F1 score is as follows: Accuracy = (TP + TN)/(TP + TN + FN + FP), Precision = TP/(TP + FP), Recall = TP/(TP + FN), F1 score = Accuracy × Recall × 2/(Accuracy + Recall). TP means true positive. FP means false positive. FN means false negative. TN means true negative.

## 3. Results

### 3.1. LR Model

In the training data set, the LR model was established with the presence or absence of macrosomia as the dependent variable and a total of 12 indicators from the collected medical records as independent variables. And the test level was set at 0.05. The values assigned to the variables entered into the model are shown in Table 1. The results of the LR model showed that age, pregnancy BMI, parity, HDL-C level, FT4 level, TPOAb positive, and gestational diabetes mellitus are risk factors for the development of macrosomia (*P* < 0.05). Model formula: P = exp(−10.455 + 0.166X1 + 1.410X2 + 1.340X4 + 1.175X6 − 0.172X8 + 2.311X9 + 0.781X10)/(1 + exp (−10.455 + 0.166X1 + 1.410X2 + 1.340X4 + 1.175X6 − 0.172X8 + 2.311X9 + 0.781X10))*P* is the probability of macrosomia. The specific results are shown in Table 2.

### 3.2. RF Model

**Figure 1:**
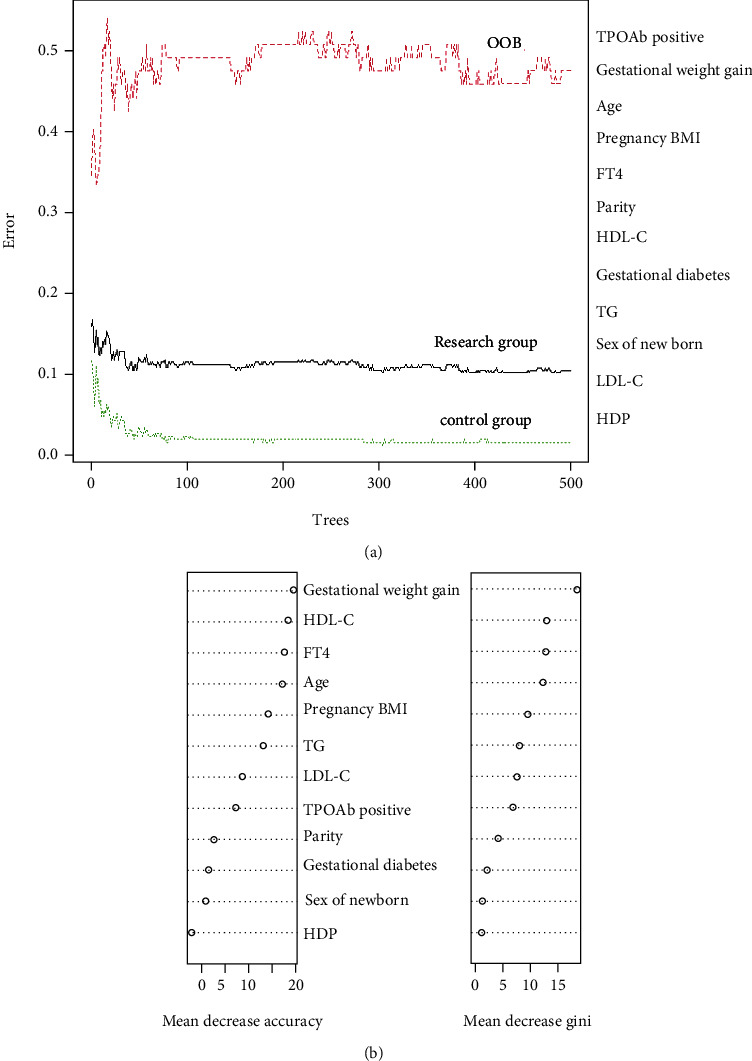
(a) OOB trend of the random forest model; (b) the classification contribution of each variable.

The constructed RF model was found to be the best model when ntree = 500 and mtry = 3. At this time, the RF model out-of-bag error rate is 6.71%, which indicates that the model generalizes well, as shown in Figure 1(a). If certain input variables have a significant effect on the outcome, then after adding noise randomly to the values of these input variables, it will have a significant effect on the output results of the outcome. The effects of each input variable on the overall prediction accuracy of the random forest model are shown in Figure 1(b). The indicators of TPOAb positive, gestational weight gain, pregnancy BMI, and FT4 level had a greater impact on the RF model accuracy, suggesting that these indicators may have a greater clinical significance, as shown in Table 3.

### 3.3. DT Model

**Figure 2:**
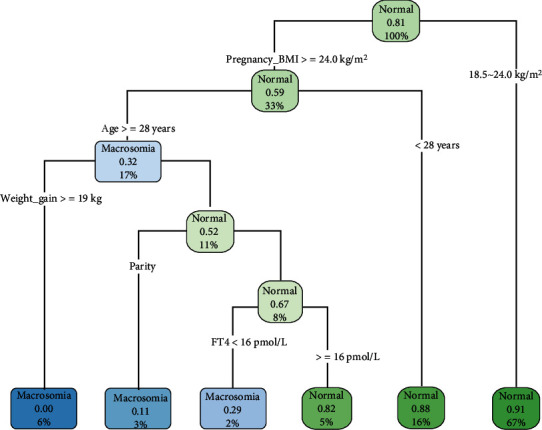
Decision tree model of macrosomia.

With the above 12 variables as predictors, the DT model was constructed by R language Rpart software package, as shown in Figure 2. The DT analysis identified 6 judgment rules, including 3 judgment rules that do not occur and 3 judgment rules that occur.

### 3.4. Performance Comparison of the Three Prediction Models

**Figure 3:**
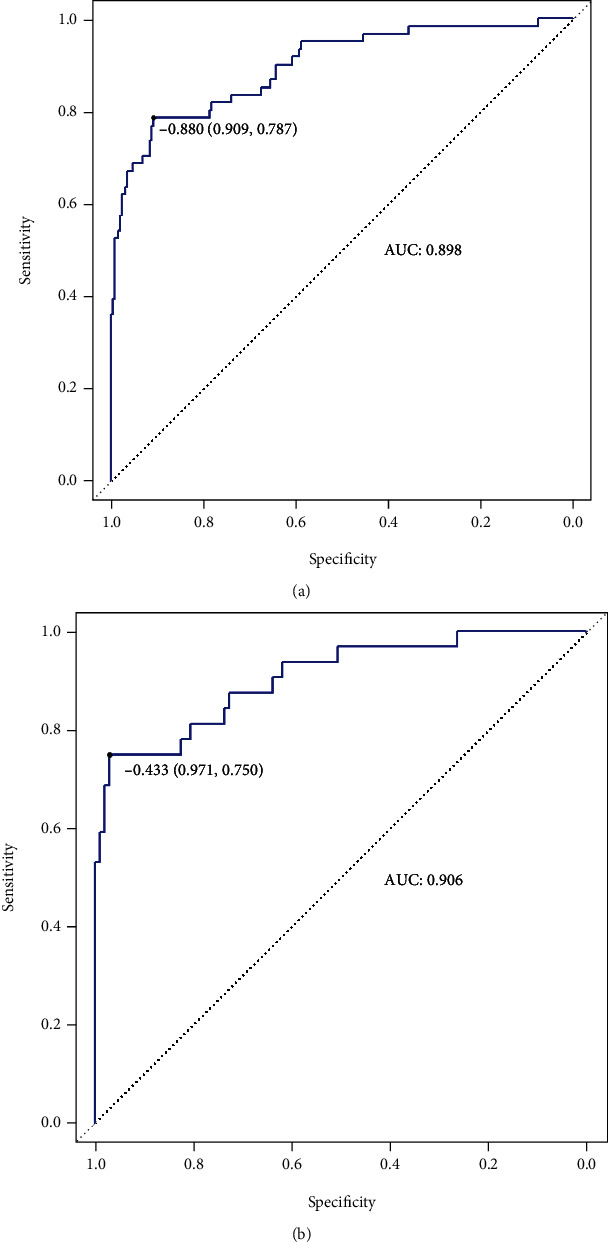
ROC curve of the logistic regression model. (a) ROC curve of training set; (b) ROC curve of validation set.

**Figure 4:**
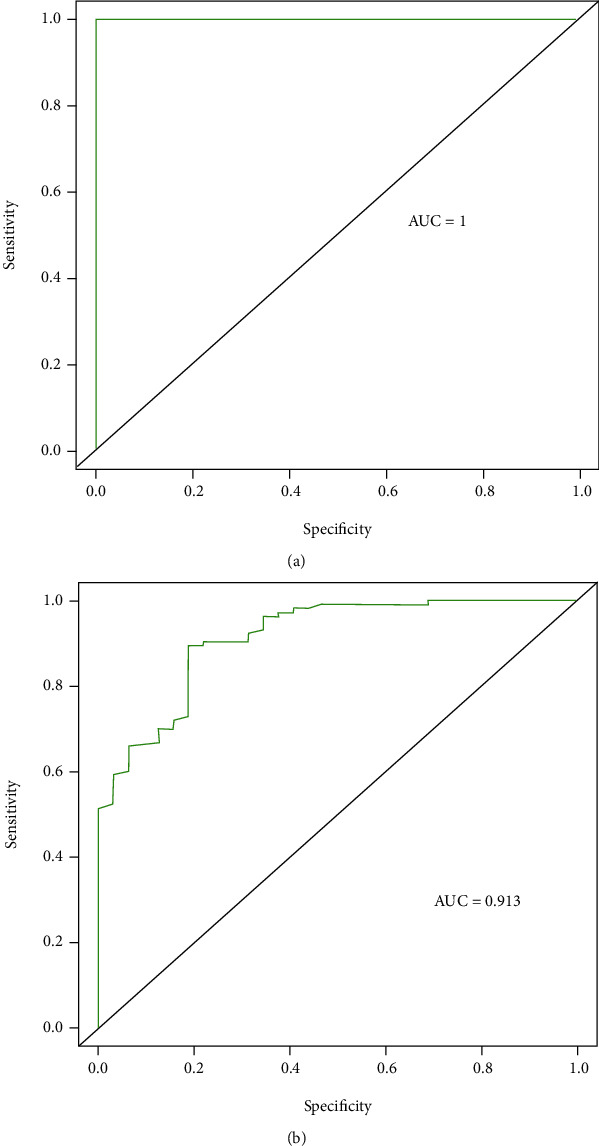
ROC curve of the random forest model. (a) ROC curve of training set; (b) ROC curve of validation set.

**Figure 5:**
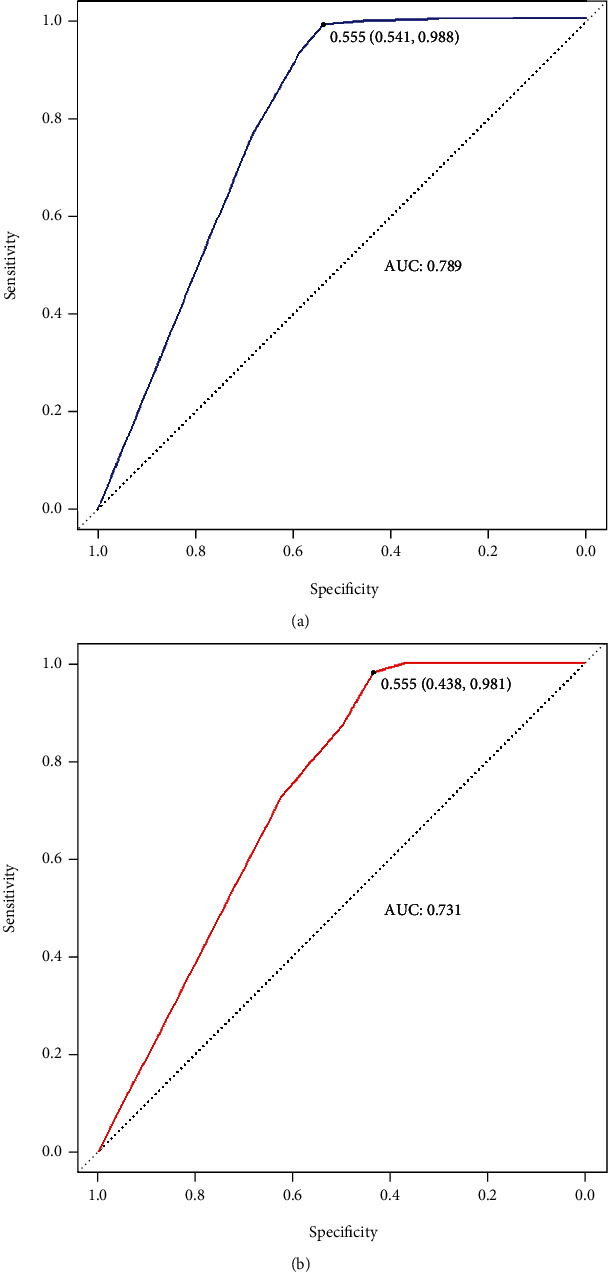
ROC curve of the decision tree model. (a) ROC curve of training set; (b) ROC curve of validation set.

In the training set, the RF model performs best with 100% accuracy. In the validation set overall, the LR model performed better with the highest accuracy, sensitivity, recall, and F1 score value (see Table 4). Overall, the LR model had the highest diagnostic performance, followed by the RF model. The ROC curves of each model are shown in Figures 3–5, respectively.

## 4. Discussion

Massive infants increase the rate of cesarean delivery and perinatal complications and have been recognized as a major cause of maternal and neonatal mortality. Currently, in clinical practice, prediction of macrosomia mainly relies on imaging measurements and obstetric examination to measure uterine height, but the accuracy of these two methods is still controversial [7–9, 13, 14]. In the study of constructing statistical models for predicting macrosomia, imaging measurement indicators and traditional data analysis methods are still dominant [15, 16]. And prediction models based on factors related to demographic characteristics, clinical data, and biochemical indicators are rarely seen. Research on prediction models for huge children based on machine learning methods still has a broad research prospect. In this paper, we collected information from hospitalized maternal medical records by a retrospective approach, screened variables from the medical records, and applied three algorithms, RF, DT, and LR, to construct different prediction models, and the comprehensive results showed that age, pregnancy BMI, gestational weight gain, FT4 level, TPOAb positive, and gestational diabetes were risk factors for the occurrence of macrosomia. This is consistent with the results reported in the literature [17]. The comparison of the performance of the three prediction models in terms of training and validation sets showed that the LR model and the RF model performed better and had a higher application in the assessment of predicting the risk of the development of macrosomia.

Said and Manji [18] reported that advanced maternal age is a risk factor for the occurrence of macrosomia, which is consistent with the results of this study. It is attributed to the current opening of the three-child policy in China and the increase in the number of third-trimester mothers of advanced maternal age. GDM increases the incidence of macrosomia due to the accumulation of fat and glucose in the fetus as a result of maternal lipid metabolism disorders. This is consistent with the study of Kansu-Celik et al. [19].

The guidelines for macrosomia issued by the American College of Obstetricians and Gynecologists [1] also point out that overweight or obesity before pregnancy, excessive weight gain during pregnancy, and gestational diabetes increase the incidence of macrosomia. Alberico et al. [20] found that in Italy, prepregnancy thinness reduced the risk of delivering a large child by 50%, and the risk of delivering a large child with prepregnancy obesity was 1.7 times higher than that of a pregnant woman with normal prepregnancy body mass. Enomoto et al. [21] found that prepregnancy thinness reduced the risk of delivering a large child by 62%, and prepregnancy overweight and obesity increased the risk of delivering a large child by 1.6 and 3.6 times. Our study suggests that maternal prepregnancy overweight and excessive prepregnancy weight gain are risk factors for the development of macrosomia. This finding is consistent with the findings of several authors [20–24]. Due to the excessive accumulation of body fat before pregnancy in overweight or obese women and excessive prenatal waist circumference growth and alteration of maternal endocrine hormone levels combined with excessive attention to nutrition and fetal preservation after pregnancy, this makes overnutrition during pregnancy lead to an increase in the occurrence of huge babies year by year.

Prepregnancy thyroid function is related to the occurrence of macrosomia, and FT4 has an impact on fetal development. We also found that maternal FT4 levels and positive TPOAb are also associated with the development of macrosomia. A study of 6031 Chinese pregnant women showed that low FT4 levels during pregnancy was a risk factor for gestational diabetes and preeclampsia [25]. Maternal thyroid function in early pregnancy is associated with fetal growth, and FT4 in pregnant women during pregnancy is negatively associated with birth weight [26].

In pregnant women, large babies are associated with prolonged labor and delivery, increased rates of vaginal obstruction and cesarean section, and a range of complications including postpartum hemorrhage. The fetus is also at increased risk for shoulder dystocia, brachial plexus injury, hypoglycemia, and even asphyxia, as well as for adult health status, including obesity and cardiovascular disease [4, 5, 27]. Therefore, early detection of high-risk pregnant women and timely pregnancy care and guidance are of great importance to prevent the development of huge babies and promote maternal and infant health.

In this paper, we collected inpatient maternal medical records through a retrospective method, screened variables from the medical records, and applied three algorithms, RF, DT, and LR, to construct different prognostic models. Through comparative study, we found that the predictive performance of the RF algorithm model in the training set was higher than that of the LR model and DT model, showing good advantages with accuracy and accuracy rate of 100%. This may be related to the fact that the randomized feature selection idea of the RF algorithm performs better than other classifiers (e.g., discriminant analysis, support vector machines, and neural networks) and it is also very comfortable in dealing with the overfitting problem [28]. The RF algorithm is an emerging-integrated machine learning algorithm with high noise immunity and stable performance, and has been widely used in the fields of risk assessment and risk factor exploration for chronic diseases, especially cardiovascular diseases [29, 30]. However, this algorithm cannot explain the direction of action and relative risk of independent variables, while LR models can explain the model and variables better. When performing data analysis, the variable magnitudes, outliers, and biased distributions have little effect on the DT model, but there are cases where the way the DT model handles numerical input variables can result in the loss of valuable data. In addition, the model parameters, the ratio of training and test data sets, and the handling of unreasonable data have some influence on the comprehensive performance of the DT model. However, in the validation set, the LR model performed better with the highest accuracy, sensitivity, recall, precision, and F1 score values. Therefore, based on the results of the comparison of the diagnostic effects of the models, both LR models and RF models can assist clinicians in early prediction of the risk of occurrence of macrosomia, and RF models can be used as a supplement to LR prediction models.

## 5. Conclusion

In conclusion, age, pregnancy BMI, gestational weight gain, FT4 level, TPOAb positive, and gestational diabetes are risk factors for macrosomia, and LR models and RF models have better results in predicting the risk of macrosomia. Therefore, it is recommended to combine the advantages of both in future studies and applications to make them better in predicting the risk of macrosomia. Limitations of the study: (1) the survey population was fixed hospital patients, and selection bias could not be avoided. (2) The medical record information was mostly secondary data, and factors such as differences in medical records of different patients and entry problems of subject members often led to uneven data quality, resulting in poor model construction. (3) Unstructured data were not fully utilized in the model construction process. The unstructured data in medical records mainly include image data such as images and slices generated during the treatment of inpatients and also include textual data such as patients' complaints, patient care conditions, and attending physicians' opinions. (4) Dietary condition variables and living environment variables were not quantified in detail, which may affect the accuracy of the model. In response to the above problems, we will address each of them in the follow-up and continue to incorporate more variables for in-depth study.

## Figures and Tables

**Table 1:** Assignment table of each input variable.

Variable	Assignment	Number
Age	Actual value entry	X1
Pregnancy BMI (kg/m^2^)	0 = Normal, 1 = overweight/obese	X2
Gestational weight gain (kg)	Actual value entry	X3
Number of maternity	0 = Primipara, 1 = parity	X4
TG (mmol/L)	Actual value entry	X5
HDL-C (mmol/L)	Actual value entry	X6
LDL-C (mmol/L)	Actual value entry	X7
FT4 (pmol/L)	Actual value entry	X8
TPOAb positive	0 = no, 1 = yes	X9
Gestational diabetes	0 = no, 1 = yes	X10
HDP	0 = no, 1 = yes	X11
Sex of newborn	0 = female, 1 = male	X12

**Table 2:** . LR model results.

Variable	Coeff	Standard error	*Z* value	*P* value
Constant	-10.455	1.890	-5.50	<0.05
Age	0.166	0.037	4.46	<0.05
Pregnancy overweight/obese	1.410	0.267	5.29	<0.05
Gestational weight gain	0.074	0.042	1.77	>0.05
Number of maternity	1.340	0.388	3.45	<0.05
TG	0.153	0.142	1.08	>0.05
HDL-C	1.175	0.500	2.35	<0.05
LDL-C	-0.046	0.261	-0.17	>0.05
FT4	-0.172	0.043	-4.00	<0.05
TPOAb positive	2.311	0392	5.90	<0.05
Gestational diabetes	0.879	0.393	2.23	<0.05
HDP	0.781	0.424	1.84	>0.05
Sex of newborn	0.478	0.359	1.33	>0.05

**Table 3:** The contribution of each variable.

Number	Variable	Mean decrease accuracy	Mean decrease Gini
1	TPOAb positive	19.608	6.953
2	Gestational weight gain	18.486	1.619
3	Age	17.662	12.293
4	Pregnancy BMI	17.115	9.660
5	FT4	14.323	12.865
6	Parity	13.260	4.214
7	HDL-C	8.498	13.020
8	Gestational diabetes	7.498	2.142
9	TG	2.357	8.094
10	Sex of newborn	1.509	1.282
11	LDL-C	0.658	7.573
12	HDP	-2.248	1.161

**Table 4:** Performance comparison of the three prediction models.

Models	LR		RF		DT	
	Training set	Validation set	Training set	Validation set	Training set	Validation set
Accuracy	0.904	0.926	1.000	0.582	0.901	0.852
Sensitivity	0.968	0.990	1.000	0.406	0.541	0.438
Specificity	0.639	0.719	1.000	0.990	0.988	0.981
Recall rate	0.968	0.990	1.000	0.406	0.541	0.438
Accurate rate	0.918	0.919	1.000	0.929	0.917	0.875
F1 score	0.942	0.953	1.000	0.565	0.680	0.583
AUC	0.898	0.906	1.000	0.913	0.789	0.731

## Data Availability

The data used to support the findings of this study are available from the corresponding author upon request.
